# The transcriptomic fingerprint of glucoamylase over-expression in *Aspergillus niger*

**DOI:** 10.1186/1471-2164-13-701

**Published:** 2012-12-13

**Authors:** Min Jin Kwon, Thomas R Jørgensen, Benjamin M Nitsche, Mark Arentshorst, Joohae Park, Arthur FJ Ram, Vera Meyer

**Affiliations:** 1 Department Molecular Microbiology and Biotechnology, Institute of Biology Leiden, Leiden University, Sylviusweg 72, 2333 BE, Leiden, The Netherlands; 2 Kluyver Centre for Genomics of Industrial Fermentation, P.O. Box 5057 2600 GA, Delft, The Netherlands; 3 Novo Nordisk, Protein Expression, 2760, Måløv, Denmark; 4 Institute of Biotechnology, Department Applied and Molecular Microbiology, Berlin University of Technology, Gustav-Meyer-Allee 25, 13355, Berlin, Germany

**Keywords:** Aspergillus niger, Protein expression, Secretion, HacA, Unfolded protein response, Endoplasmic reticulum, Glucoamylase, Transcriptome

## Abstract

**Background:**

Filamentous fungi such as *Aspergillus niger* are well known for their exceptionally high capacity for secretion of proteins, organic acids, and secondary metabolites and they are therefore used in biotechnology as versatile microbial production platforms. However, system-wide insights into their metabolic and secretory capacities are sparse and rational strain improvement approaches are therefore limited. In order to gain a genome-wide view on the transcriptional regulation of the protein secretory pathway of *A. niger*, we investigated the transcriptome of *A. niger* when it was forced to overexpression the *glaA* gene (encoding glucoamylase, GlaA) and secrete GlaA to high level.

**Results:**

An *A. niger* wild-type strain and a GlaA over-expressing strain, containing multiple copies of the *glaA* gene, were cultivated under maltose-limited chemostat conditions (specific growth rate 0.1 h^-1^). Elevated *glaA* mRNA and extracellular GlaA levels in the over-expressing strain were accompanied by elevated transcript levels from 772 genes and lowered transcript levels from 815 genes when compared to the wild-type strain. Using GO term enrichment analysis, four higher-order categories were identified in the up-regulated gene set: i) endoplasmic reticulum (ER) membrane translocation, ii) protein glycosylation, iii) vesicle transport, and iv) ion homeostasis. Among these, about 130 genes had predicted functions for the passage of proteins through the ER and those genes included target genes of the HacA transcription factor that mediates the unfolded protein response (UPR), e.g. *bipA, clxA, prpA, tigA* and *pdiA*. In order to identify those genes that are important for high-level secretion of proteins by *A. niger*, we compared the transcriptome of the GlaA overexpression strain of *A. niger* with six other relevant transcriptomes of *A. niger*. Overall, 40 genes were found to have either elevated (from 36 genes) or lowered (from 4 genes) transcript levels under all conditions that were examined, thus defining the core set of genes important for ensuring high protein traffic through the secretory pathway.

**Conclusion:**

We have defined the *A. niger* genes that respond to elevated secretion of GlaA and, furthermore, we have defined a core set of genes that appear to be involved more generally in the intensified traffic of proteins through the secretory pathway of *A. niger*. The consistent up-regulation of a gene encoding the acetyl-coenzyme A transporter suggests a possible role for transient acetylation to ensure correct folding of secreted proteins.

## Background

Due to its well annotated genome sequence, newly established gene transfer systems, and the availability of high-quality tools for obtaining and evaluating transcriptomic and proteomic data, *Aspergillus niger* has become a model fungus for industrially exploited filamentous fungi [1-4]. Its impressive natural capacity to secrete high amounts of hydrolytic proteins into the environment combined with its ability to synthesize and secrete various organic acids makes it highly suitable for the production of various food ingredients, pharmaceuticals, and industrial enzymes [1,2,5]. As it is also capable of efficiently degrading plant-derived polysaccharides such as starch, cellulose, hemicellulose, pectin, and inulin, the biotechnological importance of *A. niger* will probably rise even more in the near future. For example, *A. niger*-derived (hemi)cellulases might be used to improve the efficiency of the saccharification process of second-generation feedstock used for bioethanol production [3,6].

To analyze and eventually control the secretory capabilities of *A. niger*, several attempts have been undertaken to identify the key players and regulatory mechanisms involved in protein secretion. For example, galacturonic acid, xylose, and maltose were shown to induce expression of secretory proteins, including pectinolytic, (hemi)cellulolytic and glucan-hydrolyzing enzymes, respectively, whereas sorbitol acts as a repressing carbon source [7-10]. Xylose is the main inducer of XlnR, the master transcription factor that regulates expression of all major enzymes involved in the degradation of (hemi)cellulose [9-11]. Among those, endoxylanase (XynB) and ferulic acid esterase (FaeA) are the most abundant, secreted proteins of *A. niger*[10]. When starch or maltose are used as carbon source, the synthesis of amylolytic enzymes is induced, a step that is mediated by the transcription factor AmyR. The most abundant enzyme secreted under these conditions is a glucan 1,4-α-glucosidase (glucoamylase, GlaA), which is an exo-enzyme that releases glucose from the non-reducing end of starch or maltose and accounts for more than 50% of the extracellular proteome [10]. Induction of extracellular hydrolytic enzymes is thus mainly regulated at the transcriptional level in *A. niger*, and either repressed by carbon catabolite repression via CreA [12] or activated by AmyR or XlnR depending on the presence of maltose or xylose, respectively [13].

*A. niger* proteins and enzymes destined for secretion into the culture medium follow the secretory pathway. The journey of secretory proteins starts with protein translocation into the lumen of the endoplasmic reticulum (ER) via a translocon that forms a channel through the ER membrane [14]. In the ER lumen, several ER-resident chaperones and foldases, including the binding protein (BipA), protein disulfide isomerase (PdiA), and calnexin (ClxA), assist secretory proteins in proper folding [15]. Most secretory proteins become glycosylated in the ER, both through the attachment of a conserved, pre-assembled oligosaccharide to specific asparagine residues (*N*-glycosylation) and by the initiation of *O*-glycosylation of serine and threonine residues. After proper folding and glycosylation, secretory proteins are packed into COPII-coated vesicles and transported to the Golgi complex, where protein glycosylation is completed, and subsequently through another vesicle-mediated process delivered to the cell surface where the vesicles release their cargo into the periplasmic region. High protein flux through the ER or expression of heterologous proteins can result in the accumulation of misfolded proteins. This misfolding, however, is recognized by a quality control system known as ER-associated degradation (ERAD). The aim of ERAD is to direct misfolded proteins to the cytosol where they become degraded by the proteasome. In addition, another cellular protein quality system, the unfolded protein response (UPR), is induced, which aims at proper refolding of misfolded proteins via induced expression of chaperones and foldases [5,16]. For this purpose, the UPR transcription factor HacA becomes activated via splicing of an unconventional 20-nt intron out of the *hacA* mRNA. This subsequently facilitates translation of *hacA* mRNA and formation of HacA, which in turn induces transcription of a number of UPR target genes including *bipA*, which encodes the major ER chaperone protein, and *pdiA*, which encodes for protein disulfide isomerase [17,18]. Hence, both ERAD and UPR are crucial for effective functioning of the secretory pathway - not only in filamentous fungi but also in yeasts and mammals [19-22].

Interestingly, the UPR can be viewed as a general response of *A. niger*, which becomes activated when the secretion machinery becomes challenged by metabolic changes or ER stress conditions. For example, transcript levels of UPR genes become strongly enhanced, when *A. niger* is exposed to the reducing compound dithiothreitol (DTT), which blocks the formation of disulfide bridges, or to tunicamycin, which inhibits *N*-glycosylation, especially when forced to express heterologous proteins or when cultivated in carbon sources, which differentially induce expression and secretion of homologous proteins [18,23,24]. These observations suggest that the UPR functions as a homeostatic control mechanism that allows *A. niger* to flexibly adapt its protein secretion capacity to change in environmental conditions.

In this study, we investigated the transcriptomic fingerprint of *A. niger* when forced to overexpress and secrete a specific hydrolytic enzyme. We chose glucoamylase (GlaA) as a model enzyme, because it is a naturally highly abundant and secreted enzyme used in the food industry [25]. For comparison, we used two strains, a wild-type strain expressing a single copy of the *glaA* gene, and a mutant strain expressing multiple copies of *glaA*. In order to reduce the number of conflicting variables, such as changes in growth rates and fluctuations in environmental conditions, maltose-limited chemostat cultures were used. Maltose was selected as a carbon source to transcriptionally induce expression of the *glaA* gene. Physiological and transcriptomic data were collected from both wild-type and overexpressing strains and analyzed to identify the *glaA*-specific overexpression transcriptome. Finally, we compared these global transcriptional changes with previously published transcriptomic data related to secretion stress in *A. niger*. This analysis allowed us to distinguish condition-specific responses from general transcriptomic responses of *A. niger* that are important to overcome different triggers of secretion stress. We could identify a core set of 40 genes whose expression is key to ensure high protein fluxes through the secretory route of *A. niger*, independently of the cause of the secretion stress.

## Results and discussion

### Growth physiology of maltose-limited chemostat cultures of A. niger

In order to identify the transcriptomic adaptations of *A. niger* to forced overproduction of GlaA, we compared the transcriptomes of chemostat-grown cultures of strain B36 (overproducing strain) and wild-type strain N402 (reference strain). Strain B36 was selected as GlaA overproducer, because it is reported to contain multiple copies of the *glaA* gene at chromosome V [26,27].

**Figure 1:**
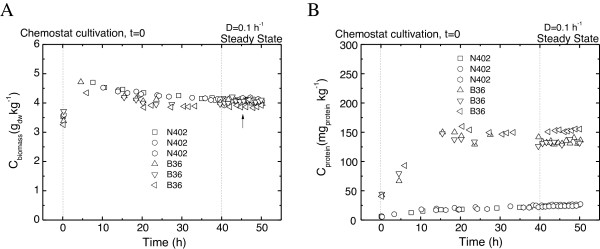
**Growth profiles (A) and extracellular protein production (B) of N402 and B36.** The growth curves presented are based on (**A**) dry-weight biomass concentration and on (**B**) total protein concentration in culture filtrates. An arrow indicates RNA sampling for transcriptomics. All six independent cultures are shown.

Maltose-limited chemostat cultures were used to induce expression of the *glaA* gene, to control the specific growth rate and to obtain highly reproducible data due to well-defined steady state conditions. Initial chemostat experiments were conducted using three different dilution rates (D = 0.05, 0.1, and 0.15 h^-1^); however, steady state conditions for both strains were only reached at D = 0.1 h^-1^. At a higher dilution rate (D = 0.15 h^-1^), N402 reached a steady state, but the B36 strain was washed out before reaching a steady state. Although the morphology of the N402 strain at the lowest dilution rate (D = 0.05 h^-1^) was similar to the higher dilution rates, and no sign of mycelial aggregation was apparent (data not shown), N402 was not able to reach a steady state at this dilution rate. Maltose-limited chemostat cultures were therefore run in triplicate at D = 0.1 h^-1^ for both strains. The cultures were highly reproducible and gave rise to homogenous cultures of dispersed mycelial morphologies (Figure 1, Table 1, and data not shown). The initial batch cultivation (duration about 30 h) was followed by approximately 50 h of continuous cultivation. After four volume changes (4 x D^-1^), the cultures reached a steady state as reflected by a constant alkali addition rate and constant CO_2_, O_2_, and biomass concentrations (Figure 1 and data not shown). Biomass concentrations for both strains stabilized at about 4 g kg^-1^ with a small relative standard deviation (RSD) (approx. 0.002 for N402 and 0.02 for B36). In both strains, the respiratory quotient (RQ) was lower than 1, probably due to high production of organic acids in parallel to GlaA secretion. Notably, the RQ value calculated for B36 cultures was slightly but significantly lower than the RQ value obtained for N402 cultures (Table 1), suggesting that protein and acid production is somewhat higher in B36 compared to N402. In agreement, the carbon concentration in culture filtrates obtained from steady state samples was also higher in B36 compared to N402 (data not shown) and the specific productivity of extracellular protein (q_protein-EC_) was five- to six-fold higher in B36 than N402, demonstrating that B36 indeed secretes more protein than the wild-type strain.

**Table 1:** Physiology of N402 and B36 in chemostat cultures

	**C**_**biomass **_**(g**_**DW **_**kg**^**-1**^**)**	**Y**_**x/s **_**(g**_**DW **_**g**_**maltose**_^**-1**^**)**	**Y**_**x/c **_**(g**_**DW **_**g**_**carbon**_^**-1**^**)**	**q**_**CO2 **_**(mmol g**^**-1**^**h**^**-1**^**)**	**q**_**O2 **_**(mmol g**^**-1**^**h**^**-1**^**)**	**RQ**	**q**_**protein-EC **_**(mg g**^**-1**^**h**^**-1**^**)**	**C-recovery (%)**
N402	4.09 ± 0.01	0.49 ± 0.02	1.22 ± 0.05	2.11 ± 0.05	2.38 ± 0.06	0.88 ± 0.03	**0.60 ± 0.03**	92 ± 0.8
B36	4.01 ± 0.09	0.47 ± 0.01	1.17 ± 0.03	2.05 ± 0.07	2.51 ± 0.14	0.82 ± 0.03	**3.49 ± 0.37**	89 ± 0.3

Steady state results of maltose-limited chemostat cultures. Mean values ± standard deviations are given for N402 and B36 from triplicate independent steady-state cultures. Bold letters indicate significant differences based on a two tailed t-test (p < 0.001). C_biomass_, dry weight of biomass concentration; Y_x/s _and Y_x/c_, growth yield on substrate and carbon; q_CO2 _and q_O2_, specific carbon dioxide evolution rate and oxygen consumption rate; RQ, respiratory quotient; q_protein-EC_, specific extracellular protein production rate; C-recovery, carbon recovery.

### Protein secretion and glucoamylase production during steady state

The GlaA overproducer strain B36 was previously estimated to contain about 80 copies of the *glaA* gene as inferred from Southern analysis [26]. As multiple gene copies can cause frequent recombination in *A. niger* resulting in genetic instability and loss of *glaA* copies [27], we decided to re-determine the number of *glaA* copies present in B36 using quantitative real time PCR (qPCR). As summarized in Table 2, B36 contains about 32 *glaA* gene copies based on the fact that N402 contains only a single copy of *glaA*[3]. We also determined *glaA* mRNA levels by qPCR and observed that *glaA* transcript levels in B36 were about seven times higher compared to N402. Consistent with this observation, under steady state conditions extracellular GlaA production in B36 was about seven- to eight-fold higher than in N402 as estimated by Western analysis and measuring glucoamylase activity (Table 2). These data confirm previous observations that the amount of GlaA produced correlates well with the amount of *glaA* mRNA but is not proportional to the number of *glaA* gene copies, a well-known phenomenon in filamentous fungi, where protein overproduction is often limited at the transcriptional level [13,27]. It should be noted, however, that the increased glucoamylase production in the B36 strain is still significantly lower compared to industrial strains which produce glucoamylase up to 30 gram/liter [28].

**Table 2:** Glucoamylase assessments in N402 (wild-type strain) and B36 (overexpressing strain)

	**N402**	**B36**
glucoamylase gene copy number^a^	1	32 ± 5
glucoamylase transcripts level^a^	1	6.9 ± 0.9
glucoamylase protein detection (g^-1^)^b^	1	8.3 ± 1.8
glucoamylase activity (U g^-1^)^c^	154 ± 68	1057 ± 398

Glucoamylase was assessed using steady-state samples. Mean values ± standard deviations for N402 and B36 were calculated from triplicate measurements of respective triplicate steady-state samples. ^a^Glucoamylase gene copy number and relative glucoamylase transcripts level were assessed by qPCR using genomic DNA and cDNA as templates. ^b^Western blot analysis of glucoamylase protein. Culture filtrate samples corrected for equal amounts of biomass were loaded onto SDS-PAGE. The amount of glucoamylase protein was represented as relative values based on N402. ^c^Glucoamylase activity was determined by measuring liberated glucose from starch.

### The GlaA-overexpression transcriptome

RNA samples for microarray analysis were taken from triplicate steady-state cultures of both strains. The average RSD of all genes expressed was about 0.06, indicating high reproducibility of all six chemostat cultures and transcript profiles. The expression of 1,587 genes out of 14,165 *A. niger* genes was changed: 772 displayed increased expression levels in the strain B36, and 815 genes were down-regulated in B36 (significance: FDR*, q* value < 0.005). Although the majority of differentially expressed genes (1,280 genes) showed fold-changes in gene expression <2, these values are considered to be significant in view of the identical culture conditions used, the identical specific growth rates of both strains during steady state conditions, and the stringent statistical analysis of the data. A comprehensive list of all differentially expressed genes is depicted in Additional file 1.

An enrichment analysis was performed to identify gene ontology (GO) terms which were over-represented in the differentially expressed gene set. We used the recently published improved GO annotation tool for *A. niger* (Fisher’s exact test Gene Ontology annotation tool, FetGOat [29]), an open source tool accessible at http://www.broadinstitute.org/fetgoat/index.html. GO terms (up- or down-regulated) with FDR values < 0.05 were defined as over-represented. Overall, 129 enriched GO terms were identified among the differentially expressed gene set, 54 of which belonged to ‘biological processes’ (BP), 63 to ‘cellular components’ (CC) and 12 to ‘molecular functions’ (MF). The corresponding network maps and gene lists are depicted in the Additional file 2 and Additional file 3.

### Predicted up- and down-regulated biological processes inferred from the GlaA-overexpression transcriptome

In order to deduce biological information out of the GO term enrichment analysis, we focused on the BP gene list and removed redundant and less detailed annotations. Among the remaining GO terms in the up-regulated gene set, the following four higher-order categories were identified: i) translocation, ii) protein glycosylation, iii) vesicle transport, and iv) ion homeostasis (see Additional file 4). The translocation category included GO terms such as ‘posttranslational protein targeting to membrane’, ‘SRP-dependent co-translational protein targeting to membrane’, ‘translocation’, and ‘protein targeting to ER’. The protein glycosylation category included the GO terms ‘related to glycosylation’, ‘protein N-linked glycosylation’, ‘oligosaccharide biosynthetic process’, ‘dolichol-linked oligosaccharide biosynthetic process’, and ‘oligosaccharide-lipid intermediate biosynthetic process’. The vesicle transport category included the GO terms ‘vesicle-mediated transport’, ‘COPII-coated vesicle budding’, ‘membrane budding’, ‘vesicle organization’, ‘vesicle coating’, ‘vesicle targeting (rough ER to cis-Golgi)’, ‘COPII vesicle coating’, and ‘retrograde vesicle-mediated transport (Golgi to ER)’. The last category, ion homeostasis, contained GO terms involved in iron, calcium, and zinc homeostasis (e.g. ‘iron homeostasis’, ‘inorganic cation homeostasis’, ‘cellular response to iron starvation’, and ‘ion transport’, see Additional file 4).

As three out of the four major categories were related to the secretory pathway, the corresponding genes lists were examined in more detail. The expression of at least 130 predicted secretory pathway genes were changed in the GlaA-overexpressing strain B36 (Table 3). Importantly, this set of genes is causatively linked to GlaA overexpression and does not depend on the growth rate or the carbon source, because both strains were in steady state at the same specific growth rate in maltose-limited chemostat cultures. Only 16 of the 130 genes were down-regulated, indicating that the capacity of the protein secretion machinery is increased compared to the wild-type situation. Among the secretory pathway-related genes, the majority of the induced genes belonged to ER-related processes, including translocation into the ER, protein folding, glycosylation, ERAD, UPR, and COPI- and COPII-mediated transport processes (Table 3). Notably, the gene with the most significantly increased transcript level in B36 was the *bipA* gene (FDR, 1.1 × 10^-7^), which is under transcriptional control of HacA [17], and encodes the main chaperone in the ER and is thus important for protein overproduction in *A. niger*[30]. The increased expression of *bipA* gene in the B36 strain was previously reported by Northern blot analysis in a shake flask culture [31]. In agreement, other important HacA-dependent ER chaperones and foldases such as *clxA*, *prpA, tigA*, and *pdiA* (Table 3) were also significantly higher expressed in B36 [17].

**Table 3:** Differential expression of genes encoding secretory pathway related proteins

**DSM code**	**DSM annotation**	**Fold-change B36/N402**	**P**	**FDR**
**Protein folding**				
An02g14800*	protein disulfide isomerase A *pdiA *– *A. niger*	1.72	5.98E-08	3.43E-06
An18g02020*	disulfide isomerase *tigA *– *A. niger*	1.89	1.61E-08	1.17E-06
An01g04600*	PDI related protein A *prpA* – *A.niger*	2.08	2.21E-09	2.40E-07
An16g07620*	strong similarity to endoplasmic reticulum oxidising protein Ero1 – *S. cerevisiae*	1.97	1.02E-08	8.15E-07
An18g04260*	similarity to secreted protein HNTME13 from patent WO9839446-A2 – *H. sapiens*	2.46	1.06E-09	1.28E-07
An18g06470*	strong similarity to DnaJ-like protein MTJ1 - *Mus musculus*	1.48	1.34E-06	3.70E-05
An05g00880*	strong similarity to dnaJ protein homolog Scj1 – *S. cerevisiae*	1.79	1.02E-07	5.04E-06
An01g08420*	strong similarity to calcium-binding protein precursor clx1p – *S. pombe*	2.42	8.96E-10	1.13E-07
An04g02020*	strong similarity to cyclophilin cypB – *A. nidulans*	1.69	1.19E-07	5.60E-06
An01g06670*	strong similarity to peptidyl-prolyl isomerase FKBP-21 – *N. crassa*	1.66	4.71E-07	1.57E-05
An11g04180*	dnaK-type molecular chaperone bipA – *A. niger*	2.32	8.63E-10	1.11E-07
An01g13220*	strong similarity to 150 kDa oxygen regulated protein ORP150 - *Rattus norvegicus*	2.26	1.34E-09	1.58E-07
**Signal recognition/cleavage**				
An04g06890	similarity to 72-kD protein of the signal recognition particle SRP72 - *Canis lupus*	1.33	1.14E-04	1.37E-03
An15g06470*	similarity to signal sequence receptor alpha chain - *Canis lupus*	1.86	4.49E-08	2.72E-06
An01g00560*	strong similarity to signal peptidase subunit Sec11 – *S. cerevisiae*	1.84	5.70E-07	1.84E-05
An16g07390*	strong similarity to endoplasmatic reticulum signal peptidase subunit Spc2 – *S. cerevisiae*	1.90	7.99E-09	6.74E-07
An09g05420*	similarity to signal peptidase subunit Spc3 – *S. cerevisiae*	1.99	6.90E-09	6.03E-07
**Translocation into ER**				
An03g04340*	strong similarity to ER membrane translocation facilitator Sec61 - *Yarrowia lipolytica*	1.68	9.76E-08	4.90E-06
An01g03820	strong similarity to ER protein-translocation complex subunit Sbh2 – *S. cerevisiae*	1.62	2.60E-06	6.33E-05
An01g11630*	strong similarity to translocation complex component Sss1 – *S. cerevisiae*	1.71	1.88E-07	8.11E-06
An02g01510*	strong similarity to component of the endoplasmic reticulum protein translocation machinery Sec62 – *S. cerevisiae*	1.57	7.50E-06	1.49E-04
An01g13070*	strong similarity to signal recognition particle receptor Sec63 – *S. cerevisiae*	2.02	2.17E-08	1.47E-06
An16g08830*	strong similarity to component of ER protein-translocation subcomplex Sec71 from patent WO9949028-A1 – *S. cerevisiae*	1.80	3.47E-08	2.17E-06
An15g01670	strong similarity to signal sequence receptor alpha subunit SRP101 - *Yarrowia lipolytica*	1.29	1.14E-04	1.37E-03
An05g00140*	similarity to signal recognition particle receptor beta chain Srp102 – *S. cerevisiae*	1.39	2.57E-05	4.16E-04
**Glycosylation**				
An02g07650	strong similarity to phosphoglucomutase pgmB – *A. nidulans*	0.80	5.29E-04	4.81E-03
An03g05940*	strong similarity to glutamine-fructose-6-phosphate transaminase Gfa1 – *S. cerevisiae*	0.66	2.20E-06	5.58E-05
An04g04990*	strong similarity to mannose-1-phosphate guanyltransferase MPG1 - *Trichoderma reesei*	1.46	1.44E-05	2.57E-04
An11g02380*	strong similarity to GTP:alpha-D-mannose-1-phosphate guanylyltransferase MPG1 - *Hypocrea jecorina*	1.35	5.47E-05	7.67E-04
**An02g08660**	strong similarity to hypothetical protein H04M03.4 - *Caenorhabditis elegans*	1.25	2.90E-04	2.91E-03
An03g06940*	strong similarity to UPD-GlcNAc transporter MNN2-2 - *Kluyveromyces lactis*	1.35	2.72E-05	4.38E-04
An02g14560*	oligosaccharyltransferase alpha subunit OstA – *A. niger*	2.05	2.27E-09	2.40E-07
An07g04190*	strong similarity to dolichyl-diphospho-oligosaccharide--protein glycosyltransferase 48kD chain DDOST - *Gallus gallus*	1.69	1.03E-07	5.04E-06
An18g03920*	strong similarity to defender against apoptotic cell death DAD1 - *Homo sapiens*	2.04	2.49E-09	2.56E-07
An02g14930*	strong similarity to dolichyl-diphosphooligosaccharide-protein glycotransferase gamma chain Ost3 - *S. cerevisiae*	1.47	1.51E-06	4.08E-05
An16g08570*	strong similarity to translation initiation factor 3 47 kDa subunit stt3p – *S. pombe*	1.78	2.74E-07	1.07E-05
An16g04330*	strong similarity to mannose phospho-dolichol synthase dpm1 - *Hypocrea jecorina*	1.63	2.72E-07	1.07E-05
An01g05200*	strong similarity to DPM2 - *Mus musculus*	1.43	4.84E-05	6.98E-04
An03g04410*	strong similarity to UDP-glucose:dolichyl-phosphate glucosyltransferase Alg5 – *S. cerevisiae*	1.78	3.47E-07	1.26E-05
An02g03240*	strong similarity to UDP-N-acetylglucosamine--dolichyl-phosphate N-acetylglucosaminephosphotransferase Alg7 – *S. cerevisiae*	1.92	1.76E-08	1.24E-06
An06g01100*	strong similarity to mannosyltransferase Alg1 – *S. cerevisiae*	1.27	1.50E-04	1.70E-03
An14g05910*	strong similarity to mannosyltransferase Alg2 – *S. cerevisiae*	1.93	2.28E-08	1.53E-06
An18g05910*	strong similarity to hypothetical glycosyl transferase SPCC330.08 – *S. pombe*	1.49	7.14E-05	9.49E-04
An02g14940*	strong similarity to human transmembrane protein HTMPN-23 from patent WO9961471-A2 - *Homo sapiens*	1.49	2.62E-06	6.38E-05
**An04g03130**	strong similarity to mannosylation protein Lec35 - *Cricetulus griseus* [putative sequencing error]	1.56	4.25E-07	1.46E-05
An18g02360*	strong similarity to Dol-P-Man dependent alpha(1–3) mannosyltransferase Alg3 – *S. cerevisiae*	1.92	1.74E-08	1.24E-06
An08g07020*	similarity to mannosyl transferase Alg9 – *S. cerevisiae*	1.48	7.80E-06	1.53E-04
An01g08460*	strong similarity to the mannosyltransferase Alg12 – *S. cerevisiae*	1.37	4.69E-04	4.36E-03
An02g12630*	strong similarity to glucosyltransferase Alg6 - *S. cerevisiae*	1.37	1.35E-05	2.44E-04
An04g08820*	strong similarity to glucosyltransferase Alg8 – *S. cerevisiae*	1.24	3.24E-04	3.22E-03
An02g02980*	strong similarity to protein influencing Itr1 expression Die2 – *S.s cerevisiae*	1.42	4.53E-05	6.58E-04
An15g01420*	strong similarity to glucosidase I Cwh41 – *S. cerevisiae*	1.81	2.90E-08	1.84E-06
An18g05620	strong similarity to glucosidase II alpha subunit AAF66685.1 - *Homo sapiens*	0.80	4.70E-04	4.37E-03
An01g10930*	strong similarity to enzyme with sugar transferase activity from patent JP11009276-A - *Acremonium sp.*	0.45	2.27E-09	2.40E-07
An04g06920*	extracellular alpha-glucosidase aglU - *Aspergillus niger*	0.60	7.55E-08	4.07E-06
**An09g03300**	strong similarity to alpha-xylosidase XylS - *Sulfolobus solfataricus*	0.80	4.19E-04	3.98E-03
An09g05880*	strong similarity to alpha-glucosidase ModA - *Dictyostelium discoideum*	1.75	4.77E-08	2.86E-06
An13g00620*	strong similarity to 80K protein H precursor G19P1 - *Homo sapiens*	1.53	1.24E-06	3.47E-05
An07g06430*	strong similarity to glycoprotein glucosyltransferase gpt1p – *S. pombe*	1.64	3.96E-07	1.39E-05
An01g12550	strong similarity to mannosyl-oligosaccharide 1,2-alpha-mannosidase msdS – *A. saitoi*	0.32	2.29E-11	8.76E-09
An06g01510	strong similarity to class I alpha-mannosidase AAB62720.1 - *Spodoptera frugiperda*	0.74	4.29E-05	6.31E-04
An12g00340*	similarity to alpha 1,2-mannosidase IB - *Homo sapiens*	1.54	7.61E-06	1.50E-04
An05g01750	strong similarity to alpha-1,6-mannosyltransferase Hoc1 – *S. cerevisiae*	0.52	1.04E-08	8.30E-07
An11g07490	similarity to alpha-1,6-mannosyltransferase Hoc1 – *S. cerevisiae*	0.66	8.04E-07	2.46E-05
An15g03330	strong similarity to galactosyltransferase Bed1 – *S. cerevisiae*	1.42	9.62E-05	1.20E-03
An11g09890*	strong similarity to mannosyltransferase 1 PMT1 - *Candida albicans*	1.36	1.27E-04	1.49E-03
An07g10350*	protein O-mannosyl transferase pmtA – *A. niger*	1.50	4.45E-06	9.69E-05
An16g08490*	strong similarity to dolichyl-phosphate-D-mannose--protein O-mannosyltransferase Pmt4 – *S. cerevisiae*	1.49	1.89E-06	4.90E-05
**An15g04810**	similarity to alpha-1,3-mannosyltransferase Mnt2 - *S. cerevisiae*	0.75	2.96E-05	4.66E-04
**An02g11720**	strong similarity to alpha-mannosidase msd2 – *A. nidulans*	0.71	1.64E-05	2.85E-04
An01g06500	strong similarity to filamentous growth protein Dfg5 – *S. cerevisiae*	0.58	1.44E-05	2.56E-04
An02g02660	strong similarity to hypothetical protein Dcw1 – *S. cerevisiae*	0.78	2.74E-04	2.79E-03
An11g01240*	similarity to filamentous growth protein Dfg5 – *S. cerevisiae*	2.17	1.48E-08	1.10E-06
**Protein misfolding (UPR and ERAD associated degradation)**				
An08g00830	strong similarity to protein phosphatase type 2C Ptc2 – *S. cerevisiae*	1.31	5.07E-04	4.65E-03
An11g11250*	strong similarity to interferon-induced double-stranded RNA-activated protein kinase inhibitor P58 - *Homo sapiens*	1.80	2.58E-07	1.03E-05
An01g14100*	weak similarity to stress protein Herp - *Mus musculus*	1.61	1.12E-06	3.21E-05
An03g04340*	strong similarity to ER membrane translocation facilitator Sec61 – *Yarrowia lipolytica*	1.68	9.76E-08	4.9E-06
An04g00360*	strong similarity to transport vesicle formation protein Sec13 – *S. cerevisiae*	1.84	1.28E-08	9.9E-07
An15g00640*	strong similarity to hypothetical protein GABA-A receptor epsilon subunit – *Caenorhabditis elegans*	2.03	6.20E-08	3.53E-06
An16g07970*	similarity to autocrine motility factor receptor Amfr – *Mus musculus*	1.60	1.09E-05	2.02E-04
An12g00340*	similarity to alpha 1,2-mannosidase IB - *Homo sapiens*	1.54	7.61E-06	1.50E-04
An15g01420*	strong similarity to glucosidase I Cwh41 – *S. cerevisiae*	1.81	2.90E-08	1.84E-06
An18g06220*	strong similarity to alpha-mannosidase Mns1 – *S. cerevisiae*	2.10	3.47E-06	8.05E-05
An01g12720*	similarity to tumour suppressor protein TSA305 from patent WO9928457-A1 - *Homo sapiens*	1.69	3.91E-07	1.38E-05
**Protein complex involved in protein transport**				
An01g03190	similarity to protein Sec3 – *S. cerevisiae*	1.30	7.93E-05	1.03E-03
An08g05570*	similarity to secretory protein Sec5 – *S. cerevisiae*	1.54	1.97E-06	5.07E-05
An04g06180	strong similarity to exocyst subunit Sec6 – *S.s cerevisiae*	1.38	7.87E-05	1.03E-03
An08g07370	similarity to exocyst protein Exo84 – *S. cerevisiae*	1.42	9.83E-05	1.22E-03
An02g14400*	strong similarity to hypothetical protein SPCC338.13 – *S. pombe*	1.43	8.59E-06	1.66E-04
An16g01630	strong similarity to enoyl reductase of the lovastatin biosynthesis lovC – *A.terreus*	0.35	1.66E-10	3.53E-08
An04g08690*	similarity to polynucleotide sequence SEQ ID NO:3913 from patent WO200058473-A2 - *Homo sapiens*	1.37	7.88E-05	1.03E-03
An02g07090*	strong similarity to ASNA1 product arsenite translocating ATPase - *Homo sapiens*	1.30	1.46E-04	1.66E-03
An01g14250*	strong similarity to delta subunit of the coatomer delta-coat protein CopD - *Bos taurus*	1.46	3.66E-06	8.35E-05
An08g01250*	weak similarity to COP1-interacting protein 7 CIP7 - *Arabidopsis thaliana*	1.75	4.69E-07	1.57E-05
An16g05370*	similarity to zinc-finger protein Glo3 - *S. cerevisiae*	1.59	1.28E-05	2.34E-04
An16g02460*	strong similarity to alpha subunit of the coatomer complex Ret1 – *S. cerevisiae*	1.67	2.40E-07	9.68E-06
An01g14260*	strong similarity to delta subunit of the coatomer delta-coat protein CopD - *Bos taurus* [deleted ORF]	1.51	1.15E-06	3.27E-05
An12g04830*	strong similarity to coatomer protein zeta chain Ret3 – *S. cerevisiae*	1.47	7.15E-06	1.44E-04
An07g06030*	strong similarity to coatomer gamma subunit 2 copg2 - *Homo sapiens*	1.65	4.32E-06	9.45E-05
An02g05870*	strong similarity to coatomer beta subunit copB2 - *Homo sapiens* [putative frameshift]	1.50	1.17E-04	1.40E-03
An01g04040*	secretion-associated GTP-binding protein sarA – *A. niger*	1.27	1.99E-04	2.13E-03
An08g03270*	strong similarity to beta-COP Sec26 – *S. cerevisiae*	1.48	2.84E-06	6.82E-05
An04g00360*	strong similarity to transport vesicle formation protein Sec13 – *S. cerevisiae*	1.84	1.28E-08	9.90E-07
An02g01690*	strong similarity to p150 component of the COPII coat of secretory pathway vesicles Sec31 – *S. cerevisiae*	1.60	2.05E-07	8.64E-06
An01g04730*	strong similarity to secretory protein Sec23 – *S. cerevisiae*	1.62	2.83E-07	1.08E-05
An08g10650*	strong similarity to transport protein Sec24 – *S. cerevisiae*	1.55	6.97E-07	2.18E-05
An16g03320*	strong similarity to transport protein Sec24A - *Homo sapiens*	1.56	2.27E-06	5.69E-05
An15g01520*	strong similarity to multidomain vesicle coat protein Sec16 – *S. cerevisiae*	1.53	1.74E-06	4.55E-05
**ER to Golgi and intra-Golgi transport**				
An08g03590*	strong similarity to precursor of protein Emp24 – *S. cerevisiae*	1.39	7.73E-06	1.52E-04
An09g05490*	strong similarity to COP-coated vesicle membrane protein P24 homolog lbrA - *Polysphondylium pallidum*	1.42	4.30E-06	9.43E-05
An07g09160	strong similarity to pattern formation protein cni - *Drosophila melanogaster*	1.28	1.30E-04	1.52E-03
An01g08870*	strong similarity to component of COPII-coated vesicles Erv25 - *S. cerevisiae*	1.39	7.23E-06	1.45E-04
An08g03960*	strong similarity to hypothetical endoplasmic reticulum associated protein – *S. pombe*	1.55	7.36E-06	1.47E-04
An03g04940*	strong similarity to Erv41 - *S. cerevisiae*	2.12	1.20E-08	9.44E-07
An01g04320*	strong similarity to COPII vesicle coat component protein Erv46 - *S. cerevisiae*	2.12	7.61E-09	6.57E-07
**An02g02830**	strong similarity to protein RER1 - *Homo sapiens*	1.30	5.22E-05	7.42E-04
**An07g02190**	strong similarity to protein Sec7 - *S. cerevisiae*	1.44	9.50E-06	1.81E-04
An08g06780*	strong similarity to transport protein Uso1 - *S. cerevisiae*	1.52	2.93E-06	7.00E-05
An18g06440	strong similarity to COPII vesicle component Yip3 - *S. cerevisiae*	1.67	2.58E-06	6.30E-05
An04g01780*	strong similarity to hypothetical protein YAR002c-a - *S. cerevisiae*	1.54	1.03E-06	3.00E-05
An04g08830*	similarity to Golgi membrane protein Emp47 - *S. cerevisiae*	1.69	9.30E-07	2.78E-05
An02g04250*	similarity to protein p58 - *Rattus norvegicus*	1.77	2.01E-08	1.39E-06
An04g01990*	similarity to protein ZW10 homolog HZW10 - *Homo sapiens*	1.32	1.06E-04	1.29E-03
An04g06090	similarity to geranylgeranyltransferase type-II alpha chain Bet4 - *S. cerevisiae*	0.77	2.69E-04	2.75E-03
An08g00290*	strong similarity to golgin-160 related protein Rud3 - *S. cerevisiae*	1.52	1.11E-05	2.05E-04
An08g06330*	strong similarity to epsilon-COP - *Cricetulus griseus*	1.39	1.84E-05	3.14E-04
An07g07340*	strong similarity to luminal ER-protein retention receptor ERD2 - *Kluyveromyces marxianus*	1.65	2.00E-07	8.46E-06
**Other processes in the secretory pathway**				
An07g02170*	similarity to transport protein Bos1 - *S. cerevisiae*	1.91	1.65E-08	1.18E-06
An15g01380*	strong similarity to Synaptobrevin homolog v-SNARE Sec22 - *S. cerevisiae*	1.30	2.74E-04	2.78E-03
An18g02490*	strong similarity to ARF guanine-nucleotide exchange factor 2 Gea2 - *S. cerevisiae*	1.31	1.52E-04	1.71E-03
An07g08220*	strong similarity to clathrin associated epsin 2A - *Homo sapiens*	1.39	1.58E-05	2.76E-04
An02g08450*	secretory gene nsfA - *Aspergillus niger*	1.27	2.86E-04	2.89E-03
An02g14450*	secretory pathway Ca2+−ATPase pmrA - *Aspergillus niger*	1.51	3.12E-06	7.38E-05
An16g08470*	similarity to hypothetical cell growth regulator OS-9 - *Homo sapiens*	1.76	8.57E-07	2.58E-05
An02g03460*	similarity to hypothetical protein YIL041w - *S. cerevisiae*	1.31	1.49E-04	1.68E-03
**An04g02070**	strong similarity to clathrin heavy chain - *Bos taurus*	1.30	1.09E-04	1.33E-03
An06g01200*	strong similarity to endosomal protein Emp70 - *S. cerevisiae*	1.55	1.37E-06	3.77E-05
**An01g11960**	similarity to brefeldin A resistance protein Bfr1 - *S. cerevisiae*	1.44	1.27E-05	2.33E-04
An04g01950*	strong similarity to zinc-metalloprotease Ste24 - *S. cerevisiae*	1.63	1.61E-07	7.12E-06

DSM code: ORF identifier in *A. niger *CBS 513.88 genome sequence [3]. Genes in bold are also found in maltose/xylose transcriptomic comparison [23]. * Indicates genes that were also identified in strains with constitutively active *hacA*^*CA*^[36].

**Figure 2:**
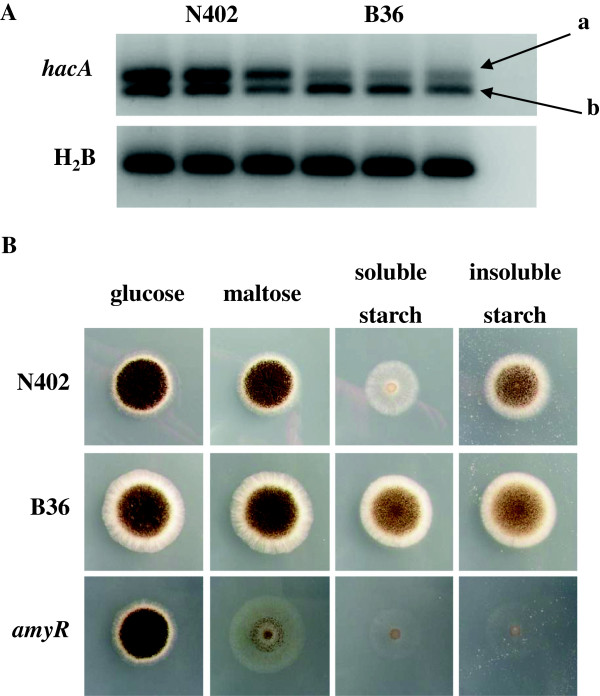
**RT-PCR analysis (A) and plate growth assay (B) of N402 and B36. A**, RT-PCR analysis of expression and transcript processing of the UPR transcription factor gene, *hacA*. The ratio between unspliced, a (220 bp), and spliced, b (200 bp), of *hacA *transcript is similar in all three N402 steady states of maltose-limited chemostat cultures while in B36 there is more spliced *hacA* present. The H2B control shows that there is no contamination with genomic DNA; genomic DNA,181-bp amplicons; mRNA, 131-bp amplicons. **B**, plate growth assay of N402 and B36 using different carbon sources. 10^4 ^spores were point-inoculated on MM plates and incubated for 4 days at 30°C.

As up-regulation of these genes pointed towards slow or aberrant folding of GlaA and thus induction of UPR in strain B36, we tested whether the central activation mechanism of UPR, the stress-induced splicing of *hacA* mRNA and thus increased expression of HacA target genes [32], was induced in strain B36. The ratio of spliced to unspliced *hacA* mRNA was indeed considerably higher in strain B36 (Figure 2A), demonstrating that forced overexpression of GlaA provokes ER stress, leading to an UPR in order to ensure proper folding and secretion of GlaA. Figure 2B shows that this response is appropriate and sufficient for *A. niger*, because strain B36 is better adapted to grow on starch compared to the wild-type strain. Interestingly, starch- and maltose-responsive genes such as the transcription factor AmyR and AmyR-dependent hydrolase genes [33] are down regulated in B36 except *glaA* (Table 4). As previously suggested, a possible explanation for the reduced expression could be the titration of the AmyR transcription factor due to the high number of *glaA* promoter copies in this strain [27]. Alternatively, the biosynthesis of an inducer through one of the enzymes under control of AmyR might be reduced. Lower inducer levels could then lead to lower levels of active AmyR transcription factor, leading to a negative feedback loop. Down-regulation of AmyR targets (except for *glaA,* Table 4) could also be explained by the RESS (repression under secretion stress) phenomenon, described for *Trichoderma reesei*[19,34] and *Arabidopsis thaliana*[35], and predicted for *A. niger*[36]. RESS is a transcriptional feedback mechanism that is activated in response to impairment of protein folding or transport and aims at lowering the protein load in the secretory route when ER stress conditions are sensed [34]. As we have observed that GlaA overexpression induces a mild UPR (Figure 2A), we propose that down-regulation of AmyR and its target genes reflects a negative feedback mechanism similar to RESS in *T. reesei*.

**Table 4:** Expression values of genes involved in starch metabolism

**Gene ID**	**Name**	**DSM annotation**	**N402**	**B36**	**Fold-change B36/N402**	**P**	**FDR**
An04g06910	*amyR*	transcription factor of starch utilization *amyR* - *Aspergillus niger*	1176	748	0.64	1.06E-06	3.06E-05
An11g03340	*aamA*	acid alpha-amylase - *Aspergillus niger*	3782	858	0.23	1.24E-09	1.48E-07
An04g06920	*agdA*	extracellular alpha-glucosidase *aglU *- *Aspergillus niger*	12052	7257	0.60	7.55E-08	4.07E-06
An01g10930	*agdB*	strong similarity to enzyme with sugar transferase activity from patent JP11009276-A - *Acremonium sp.*	5808	2634	0.45	2.27E-09	2.40E-07
An03g06550	*glaA*	glucan 1,4-alpha-glucosidase *glaA *- *Aspergillus niger*	21376	26346	1.23^*^	3.94E-04	3.80E-03
An04g06930	*amyC*	strong similarity to extracellular alpha-amylase a*myA/amyB - Aspergillus niger*	529	288	0.54	7.68E-08	4.13E-06
An04g06920	*aglU*	extracellular alpha-glucosidase *aglU - Aspergillus niger*	12052	7257	0.60	7.55E-08	4.07E-06
An09g03100	*amyA*	strong similarity to alpha-amylase precursor *amy *- *Aspergillus niger*	359	115	0.32	7.34E-08	3.99E-06

^*^Fold difference in *glaA *is under-estimated due to the saturation of array signals. Transcript level of *glaA *in B36 was about 7-fold higher than N402 based on qPCR results.

Notably, enriched GO terms of the ‘ion homeostasis’ category indicated an increased demand for iron, calcium, and zinc. In the context of increased fluxes of GlaA through the secretory pathway in strain B36, several explanations are conceivable. First, the mature GlaA protein contains nine cysteine residues, eight of which are involved in disulfide bridge formation [37,38]. Hence, in B36 there is an increased demand for disulfide bond formation, which requires increased activity of protein disulfide isomerases. Increased amounts of PdiA, however, might result in sequestration of zinc ions in the ER [38,39], thus preventing it from binding to other proteins and causing a cellular shortage of zinc. Second, chaperones such as ClxA needs calcium as co-factor [40], hence enhanced expression of *clxA* in B36 calls for higher calcium concentrations in the ER. Third, calcium is of general importance for vesicle fusions and the function of the ER and Golgi [41,42]. Hence, the cells have to mobilize calcium from internal or external stores to ensure higher fluxes through the secretory pathway in B36. Finally, the activity of GlaA is known to be positively affected by the presence of Mn^2+^, Ca^2+^, and Fe^2+^ ions [25], which are also required for many other protein activities including heme or iron-sulfur-cluster (Fe-S) proteins [42-44]. In this context, it is interesting to note that protein secretion in *A. fumigatus* has most recently been shown to require controlled uptake of iron. Basically, the transcription factor PrtT not only positively regulates expression of protease genes but also strengthens expression of iron uptake genes [45]. We thus compared the published expression data of iron uptake genes in *A. fumigatus* (wt *versus* Δ*prtT*) with the expression data of their predicted orthologs in *A. niger* (B36 *versus* N402). Twelve out of 15 iron-uptake genes showed similar expression profiles (Additional file 5), i.e. their up-regulation in wt *versus* Δ*prtT* was mirrored in B36 *versus* N402. In agreement, expression profiles of the main iron transcription factors also matched, i.e. up-regulation/down-regulation of the activator HapX/repressor SreA in wt *versus* Δ*prtT* were comparably up-/down-regulated in B36 *versus* N402. Hence, both independent observations from two *Aspergilli* strongly indicate that proper function of the protein secretion machinery and high fluxes through the secretory pathway mandate optimal iron supply and assuring proper ion homeostasis.

GO enrichment analysis of the down-regulated gene set in B36 uncovered three major categories: i) ‘carbon catabolism’, ii) ‘amino acid catabolism’, and iii) ‘response to oxidative stress’ (Additional file 3). The first two categories might be causatively linked to the RESS phenomenon discussed above, i.e. increased GlaA secretion is only possible at the cost of other secreted proteins. As AmyR targets related to starch degradation are down-regulated in B36 (see Table 4), processes related to polysaccharide degradation have consequently to be down-regulated as well. In a recent study, 19 proteases of *A. niger* were identified in the extracellular medium, when the strain was cultivated under sorbitol-, galacturonic acid- or carbon-starvation conditions [8]. Expression of 12 of them was reduced in B36 compared to N402, two of the genes were up-regulated and expression of five genes was unaltered (Additional file 6). Hence, overall reduction in protease activity in B36 will in turn decelerate amino acid catabolic processes. An additional explanation for reduced expression of carbon and amino acid catabolic processes is that many enzymes involved in these processes are iron-dependent. Iron deficiency has been shown to trigger a metabolic response in *Saccharomyces cerevisiae* or *Schizosaccharomyces pombe*, which includes down-regulation of enzymes involved in carbon and amino acid metabolism and respiration [43,46-48]. Assuming that this also holds true for *A. niger*, reduction in catabolic processes would lower the fluxes into the citric acid cycle and into the respiratory chain, which in turn would also lower the amount of radical oxygen species produced. Hence, oxidative stress would be diminished, which in turn would result in down-regulation of oxidative stress genes as observed in B36. A slight but significantly reduced RQ in B36 compared to N402 (Table 1) supports this hypothesis. Taken together, the enriched GO term set of down-regulated genes in B36 can hypothetically be viewed as a consequence of the RESS phenomenon and reduced iron availability due to increased GlaA secretion.

Two *A. niger* proteins lacking an N-terminal signal peptide for entering the secretory route were recently determined to be abundantly present in the secretome of *A. niger*[8]. It was thus proposed that proteins can also be exported out of the cell by the so-called non-classical secretion pathway [8]. Interestingly, transcript levels of both protein-encoding genes were lowered in B36 *versus* N402 (An01g09980, fold-change B36/N402 = 0.15, FDR = 8.65 x10^-9^; An01g00370, fold-change = 0.54, FDR = 0.0001). This could mean that an accelerated activity of the classical secretory machinery is only possible at the expense of the non-classical secretion pathway – which could be another potential example for the existence of the RESS regulatory feedback loop in *A. niger*.

### Comparison of the GlaA overexpression transcriptome with the maltose transcriptome

**Figure 3:**
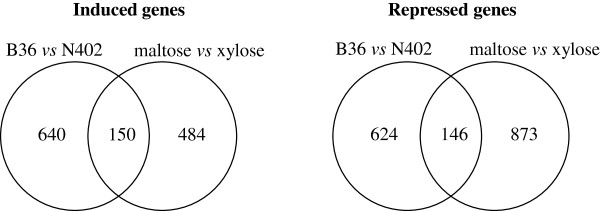
**Venn diagrams of the number of overlapping and non-overlapping induced and repressed genes on B36/N402 and maltose/xylose **[23]** chemostat cultures.**

In order to identify genes whose transcript levels are generally important for high-level secretion in *A. niger*, i.e. independent of the conditional trigger, we compared the GlaA transcriptomic fingerprint with two other recently determined fingerprints from our laboratory. First, we compared the GlaA overexpression transcriptome with the maltose transcriptome of *A. niger*. The maltose transcriptome was recently obtained by comparing carbon-limited chemostat cultures supplied with different carbon sources (maltose and xylose) but with the same specific growth rate (D = 0.16 h^-1^) [23]. This comparison showed that the production rate of extracellular proteins was about three-fold higher in maltose-grown cultures compared to xylose [23]. As shown in the Venn diagram depicted in Figure 3, 150 genes were commonly induced in both B36 *versus* N402 and maltose *versus* xylose and thus represent the most interesting genes with respect to high-level secretion in *A. niger*. GO enrichment analysis of this set of genes confirmed that genes related to ER import, translocation, N-glycosylation, and COPII transport are of utmost importance for ensuring high protein traffic through the secretory pathway (Additional file 7).

Genes that are induced in B36 *versus* N402, but not in maltose *versus* xylose, are likely to be genes that are mainly related to GlaA overexpression per se. There are 640 such genes and GO enrichment analysis uncovered two main categories (Additional file 7), one of which is the ‘ion homeostasis’ category, as expected due to the high iron, calcium and zinc demand for GlaA overexpression (see former section). The second category involves genes involved in ‘DNA repair and DNA replication’ and could hypothetically also be linked to the iron deficiency in B36 *versus* N402, as many proteins involved in nucleotide excision repair and DNA replication are iron-dependent [43]. Genes that induced in maltose *versus* xylose but not in B36 *versus* N402 are likely to be genes, which are important for starch/maltose degradation but not for xylose catabolism. Indeed, the enriched GO categories of this gene set (484 genes) contained ‘starch metabolic processes’ and other catabolic process related to ‘carbon source oxidation’, ‘amino acid metabolism’, and ‘respiration and oxidative stress’ (Additional file 7). These are processes important for (or related to) high ATP generation during oxidative phosphorylation in the respiratory chain and do likely form the energetic basis for the observed higher secretory flux in maltose cultures compared to xylose cultures despite similar growth rates. There were 146 commonly repressed genes in B36 *versus* N402 and in maltose *versus* xylose, but only a small fraction of these genes could be assigned to enriched GO terms related to ‘saccharide catabolic processes’ (Additional file 7).

### Comparison of the GlaA overexpression transcriptome with other high-secretion transcriptomes

In order to determine so far unknown HacA targets and their involvement in UPR, we have recently determined the HacA transcriptome by expressing a constitutively active form of HacA (*hacA*^*CA*^) in *A. niger*[36]. Using bioreactor-controlled batch cultures, we compared the genome-wide expression profiles of a strain expressing *hacA*^*CA*^ with a wild-type strain (*hacA*^*WT*^) at three different time points during the exponential growth phase. At each of the three time points the up-regulated gene set of the *hacA*^*CA*^ cultures contained genes related to protein traffic through the secretory pathway, and to the UPR and ERAD response [36]. When comparing these data with the GlaA-overexpression transcriptome, one might expect a considerably overlap due to the fact that overexpression of GlaA induced splicing of the *hacA* mRNA (Figure 2) and thus activation of HacA. Indeed, the transcriptional response in B36 had more in common with *hacA*^*CA*^*versus hacA*^*WT*^ (578 genes) than with maltose *versus* xylose (296 genes, data not shown). Commonly induced genes in B36 *versus* N402 and *hacA*^*CA*^*versus hacA*^*WT*^ were predicted to function in ‘vesicle coating’. ‘targeting from the ER to the Golgi’, ‘N-glycosylation’, and ‘COPII transport’.

For a more condition-independent view on how the *A. niger* transcriptome ensures high-level secretion, we finally compared the transcriptomic dataset of seven independent studies performed with *A. niger*: i) the GlaA-overexpression transcriptome obtained from chemostat cultures of B36 *versus* N402 (this study), ii) the maltose-high secretion transcriptome obtained from chemostat cultures of maltose *versus* xylose [23], iii) the HacA transcriptome reflecting permanent activation of UPR and obtained from batch cultures comparing *hacA*^*CA*^*versus hacA*^*WT*^[36], and iv) three UPR stress transcriptomes obtained from *A. niger* batch cultures stressed with DTT, tunicamycin, or forced to express the heterologous protein t-PA [18].

This analysis uncovered 40 genes whose transcript levels were commonly modified under all seven secretion stress conditions: 36 genes were up-regulated, and 4 genes were down-regulated (Table 5). The genes from this set thus probably represent genes that are crucial for coping with stress conditions that target the secretion machinery. This gene set includes ER chaperones and foldases (*prpA*, *clxA*, *lhs1*, *pdiA*, *bipA*, *tigA*), genes important for translocation of secretory proteins into the ER (*sec63, sec11, sss1, spc3, sec71*), and genes important for protein glycosylation and COPII-based vesicle trafficking (Table 5). Fascinatingly, a gene encoding the predicted acetyl-coenzyme A transporter (An02g13410) was consistently up-regulated under all seven conditions. In higher eukaryotes, this protein was shown to be involved in translocation of membrane-impermeable coenzyme A from the cytosol into the ER lumen, where it is used for transient acetylation of ER-based proteins, thus improving the folding efficiency of nascent secretory proteins [49]. It becomes therefore important to examine whether the predicted *A. niger* CoA transporter fulfills the same function. Similarly, the set of core genes included six hypothetical proteins, whose precise relevance for the secretory machinery remains to be elucidated in future studies.

**Table 5:** The list of common transcriptional response from all compared conditions

**DSM code**	**DSM annotation**	**Fold-change B36/N402**	**P**	**FDR**
**Protein folding**				
An01g13220	similar to the chaperone Lhs1	2.26	1.34E-09	1.58E-07
An02g14800	Protein disulfide isomerase PdiA	1.72	5.98E-08	3.43E-06
An01g04600	Protein disulfide isomerase PrpA	2.08	2.21E-09	2.40E-07
An01g08420	calnexin ClxA	2.42	8.96E-10	1.13E-07
An11g04180	chaperone BipA	2.32	8.63E-10	1.11E-07
An16g07620	similar to ER oxidising protein Ero1	1.97	1.02E-08	8.15E-07
An18g02020	Protein disulfide isomerase TigA	1.89	1.61E-08	1.17E-06
An11g11250	similar to the chaperone P58IPK *Homo sapiens*	1.80	2.58E-07	1.03E-05
**Translocation/Signal peptidase complex**				
An01g13070	similar to ER protein-translocation complex subunit SEC63	2.02	2.17E-08	1.47E-06
An16g08830	similar to component of subcomplex SEC71	1.80	3.47E-08	2.17E-06
An01g11630	similar to translocation complex component SSS1	1.71	1.88E-07	8.11E-06
An09g05420	similar to signal peptidase subunit SPC3	1.99	6.90E-09	6.03E-07
An01g00560	similar to signal peptidase subunit SEC11	1.84	5.70E-07	1.84E-05
An15g06470	similar to signal sequence receptor α-subunit	1.86	4.49E-08	2.72E-06
**Glycosylation**				
An14g05910	similar to mannosyltransferase ALG2	1.93	2.28E-08	1.53E-06
An03g04410	similar to glucosyltransferase ALG5	1.78	3.47E-07	1.26E-05
An02g03240	similar to N-acetylglucosaminephosphotransferase ALG7	1.92	1.76E-08	1.24E-06
An07g04190	similar to glycosyltransferase WBP1	1.69	1.03E-07	5.04E-06
An02g14560	oligosaccharyltransferase alpha subunit OSTA	2.05	2.27E-09	2.40E-07
An18g03920	similar to oligosaccharyltransferase subunit OST2	2.04	2.49E-09	2.56E-07
An18g04260	similar to UDP-galactose transporter HUT1	2.46	1.06E-09	1.28E-07
An13g00620	similar to beta subunit of an ER alpha-glucosidase	1.53	1.24E-06	3.47E-05
An15g01420	similar to glucosidase I CWH41	1.81	2.90E-08	1.84E-06
An02g14940	similar to flippase RFT1	1.49	2.62E-06	6.38E-05
**Vesicle trafficking/transport**				
An03g04940	similar to COPII vesicle coat component protein ERV41	2.12	1.20E-08	9.44E-07
An01g04320	similar to COPII vesicle coat component protein ERV46	2.12	7.61E-09	6.57E-07
An02g04250	similar to ER protein P58 (lectin family) *Rattus norvegicus*	1.77	2.01E-08	1.39E-06
An08g06780	similar to ER to Golgi transport protein USO1	1.52	2.93E-06	7.00E-05
**Lipid metabolism**				
An02g13410	similar to acetyl-coenzyme A transporter AT-1	2.10	4.55E-09	4.23E-07
**Stress related**				
An12g03580	similar to glutathione S-transferase 3 MGST3 H. sapiens	1.51	1.21E-05	2.23E-04
An01g14100	weakly similar to stress protein HERP *Mus musculus*	1.61	1.12E-06	3.21E-05
**Cell cycle and DNA processing**				
An01g08170	similar to DNA repair endonuclease RAD1 S. pombe	2.05	1.62E-08	1.17E-06
**Phosphate metabolism**				
An12g01910	similar to phytase PHYA3 Aspergillus fumigatus	0.56	2.09E-07	8.76E-06
**Cell rescue. Defense and virulence**				
An18g00980	similar to membrane protein PTH11 M. grisea	0.41	1.32E-08	1.01E-06
**Unclassified**				
An08g03960	hypothetical endoplasmic reticulum associated protein	1.55	7.36E-06	1.47E-04
An08g03970	hypothetical protein	1.87	2.87E-08	1.83E-06
An07g10280	hypothetical protein	1.43	6.40E-06	1.31E-04
An09g06130	hypothetical protein	1.64	4.24E-07	1.46E-05
An18g01000	hypothetical protein	0.56	1.05E-06	3.03E-05
An13g01520	hypothetical protein	0.49	3.57E-06	8.23E-05

DSM code: ORF identifier in *A. niger *CBS 513.88 genome sequence [3]. The list of common transcriptional response from B36/N402 (this study), maltose/xylose [23], *hacA*^*CA*^*/hacA*^*WT*^[36] and ER stress with at least 2 types of protein folding stress [18].

## Conclusions

The evidence is accumulating that the secretory machinery of *A. niger* is equiped with a high inherent flexibility, which enables the fungus to dynamically respond to changes in the secretory protein load. Irrespective of whether different amounts of a homologous protein or a heterologous protein have to be accommodated and escorted, or a specific step in the secretory pathway becomes blocked, *A. niger* follows general and specific strategies to adapt to these challenges. On the one hand, the burden of high protein loads is in general dealt with increased transcript levels of genes involved in stabilizing the secretory pathway, including chaperone-encoding and foldase-encoding genes, transport genes, and UPR- and ERAD-related genes. In addition, it seems likely that for the efficient secretion of individual proteins (either overexpressed or a heterologous proteins) an additional, particular set of genes becomes up-regulated, whose expression is important to deal with the specific requirements of the respective protein. On the other hand, *A. niger* attempts to avoid exceeding the maximum capacity of the secretory machinery. In doing so, the RESS regulatory system offers a flexible way to lower transcript levels of those genes whose function is less important for growth and survival under the given circumstances.

## Methods

### Strains and inoculums

The laboratory strain *Aspergillus niger* N402 [50] was used as a reference strain and B36 [26], which contains multiple copies of the glucoamylase gene, was used as an overproducer strain. Strains were grown on solidified Complete Medium (CM) containing 1% (w v^-1^) glucose, 0.1% (w v^-1^) casamino acids and 0.5% (w v^-1^) yeast extract in addition to Minimal Medium (MM) [51]. Spore plates were incubated for four or five days at 30°C and stored for no more than three months at 4°C. Conidia were harvested from CM agar plates with a sterile detergent solution containing 0.05% (w/v) Tween 80 and 0.9% (w/v) NaCl.

### Bioreactor cultivation conditions

Maltose-limited batch and chemostat cultivations were performed as described previously for *A. niger*[23] with slight modifications. The N402 and B36 strains were both grown in triplicate in maltose-limited chemostat cultures.

### Batch cultures

Batch cultivation was initiated by inoculation of 5 kg ammonium-based minimal medium with a conidial suspension to give 10^9^ conidia L^-1^. Maltose was heat sterilized separately from the MM and the final concentration was 0.8% (w/v). Germination was induced by addition of 0.003% (w/w) yeast extract. During the first six hours of cultivation the culture was aerated (air flow = 1 L min^-1^) through the headspace of the reactor and the stirrer speed was kept low at 250 rpm to avoid loss of the hydrophobic conidia. After six hours when most conidia had germinated, air was sparged into the culture broth, mixing was intensified (750 rpm) for more efficient oxygen transfer, and 0.01% (v/v) of polypropylene glycol (PPG) was added as an antifoaming agent. The temperature was 30°C and the pH was kept constant at pH = 3 by computer-controlled addition of 2 M NaOH or 1 M HCl. Acidification of the culture broth was used as an indirect growth measurement [52]. Submerged cultivation was performed with 6.6 L BioFlo3000 bioreactors (New Brunswick Scientific, NJ, USA).

### Chemostat cultures

Continuous cultivation was started in the late-exponential growth phase, when 90 mmol of NaOH had been added to the batch culture (75% of maltose had been consumed, at a biomass concentration of about 3.5 g dry weight per kg of culture). The dilution rate (D) was set at 0.1 h^-1^. Steady-states, where the specific growth rate (μ) is equal to the dilution rate, were defined by constant alkali addition rate and constant CO_2_, O_2_ and biomass concentrations after four residence times (≈ 40 h). Samples were taken regularly to monitor growth and to determine if a steady-state had been reached. All samples were quickly frozen in liquid nitrogen. Mycelium harvested during steady-state conditions was used for micro-array analysis.

### Analysis of culture broth

Dry weight biomass concentration was determined by weighing lyophilized mycelium separated from a known mass of culture broth. Culture broth was filtered through GF/C glass microfiber filters (Whatman). The filtrate was collected and frozen for use in solute analyses. The mycelium was washed with demineralised water, rapidly frozen in liquid nitrogen, and stored at −80°C until lyophilization. Extracellular protein concentration was determined using the Quick Start Bradford Protein Assay (Bio-Rad) with BSA as a standard. The total organic carbon in the culture filtrate was measured with a Total Organic Carbon Analyzer (TOC-Vcsn; Shimadzu, Japan), using glucose as a standard.

### RNA isolation and quality control

Total RNA was extracted by modified Trizol extraction. Frozen ground mycelium (≈200 mg) was directly suspended in 800 μl Trizol reagent (Invitrogen) and vortexed vigorously for 1 min. After centrifugation for 5 min at 13000 rpm, 450 μl of the supernatant was transferred to a new tube. Chloroform (150 μl) was added and after 3 min incubation at room temperature, samples were spun down for 15 min at maximum speed. The upper aqueous phase was transferred to a new tube to which 400 μl of isopropanol was added, followed by 10 min incubation at room temperature and centrifugation for 10 min at 13000 rpm. Pellet was washed with 75% ethanol and finally precipitated in 100 μl H_2_O. RNA samples for micro-array analysis were additionally purified on NucleoSpin RNA II columns (Machery-Nagel) according to manufacturer’s instructions. RNA quantity and quality was determined on Nanodrop spectrophotometer.

### Microarray analysis

Probe synthesis and fragmentation were performed at ServiceXS (Leiden, The Netherlands) according to the GeneChip Expression Analysis Technical Manual (Affymetrix inc., 2002). DSM (Delft, The Netherlands) proprietary *A. niger* gene chips were hybridized, washed, stained and scanned as described in the GeneChip Expression Analysis Technical Manual (Affymetrix inc., 2002).

### Normalization, filtering, statistical significance, and comparisons

Transcriptomic analysis was basically performed using Bioconductor and statistical programming language R [53]. Two experimental conditions (N402 *vs* B36) were compared to each other; each condition was represented by independent triplicate cultures. Using the robust multi-array analysis (RMA) package [54], background correction, normalization and probe summarization steps were performed according to the default setting of the RMA package. Differential gene expression was evaluated by moderated t-statistics using the Limma package [55] with a threshold of the Benjamini and Hochberg (BH) False Discovery Rate (FDR) [56] at 0.005. A minimal fold-change criterion was not applied for the identification of differentially expressed genes, as fold-changes are not necessarily related to biological relevance [57,58]. Fold-changes of gene expression from N402 to B36 (B36/N402) were calculated from normalized expression values. Means of the expression values for B36 and N402 as well as classifiers for the moderated t-statistics are summarized in Additional file 1.

### Gene ontology (GO) and enrichment analysis

Controlling the FDR at q<0.05, over-represented GO terms in sets of differentially expressed genes were determined by Fisher’s exact test [59]. An improved GO annotation for the *A. niger* CBS513.88 was applied that is based on ontology mappings from *A.nidulans* FGSCA4 (http://www.broadinstitute.org/fetgoat/index.html) [29].

### Quantitative real-time PCR (qPCR)

Quantitative real-time PCR was performed as described [60] with slight modifications. After a DNAse treatment (DNA-freeTM Kit Applied Biosystems) of 10 μg of total RNA, 600 ng were used for cDNA synthesis using the iScriptTM cDNA Synthesis Kit from BioRad according to the manufactures instructions. Real time PCR was performed in PTC-200 Peltier Thermal Cycler with a Chromo4 Continuous Fluorescence detection system from Bio-Rad with a SYBRgreen mix (iQ™ SYBR® Green supermix) in a volume of 25 μl. Each reaction was carried out in triplicate with ~200 ng of cDNA and each oligonucleotide primer at 0.3 μM. Oligonucleotide primers used for real time PCR are listed in Additional file 8. Two reference genes, H2B and Cox5 were used for normalization. The PCR program was as follows: 95°C for 3 min, followed by 39 cycles of 95°C for 15 sec and 60°C for 1 min, followed by a melting curve analysis. The efficiency of each primer pair and mean Ct (threshold cycles) values were calculated and used for determination of glucoamylase RNA transcript levels [61]. Estimation of glucoamylase gene copy number was also performed by qPCR as well but with genomic DNA as a template.

### Analysis of glucoamylase activity

One unit of glucoamylase activity was expressed as the amount of enzyme that liberates one micromole of glucose per minute from starch. Filtrates from steady-state cultures were mixed with 1% soluble starch (Sigma, S9765) in 50 mM NaAc buffer at pH 4.5 and incubated at 37°C for 3.75 min. Liberated glucose was determined with the glucose kit from ABX (Pentra Glucose HK CP).

### Western blot analysis of glucoamylase

Protein concentrations of the samples were determined with the Bradford assay using BSA as a standard. For each sample, culture filtrate samples corrected for equal amounts of biomass were mixed with 2x loading buffer (0.5 M HCl, 25% glycerol, 10% SDS, 0.5% bromophenol blue, 5% β-mercaptoethanol) and boiled for 5 min at 95°C. Protein samples were loaded on 9% SDS-PAGE gels and blotted to a nitrocellulose membrane through semi-dry electrotransfer. The membrane was blocked for 1 h with 5% low-fat dried milk in TPBS (PBS, 0.05% Tween20) and glucoamylase protein was detected using a glucoamylase-specific primary antibody (1:3,000) for 1 h at room temperature, followed by a goat anti-mouse-HRP secondary antibody (1:20,000) for 1 h at room temperature. Detection was performed using a chemiluminescence kit (Bio-Rad) according to the manufacturer’s instructions. Analysis and quantification of band intensities were performed using Image J software based on N402 signal as one.

## Competing interests

The authors declare that they have no competing interests.

## Authors’ contributions

MJK, TRJ, MA, and JP performed the chemostat experiments. MJK carried out the physiological and transcriptomic analyses and drafted the manuscript. BMN participated in initial transcriptomic analyses and the statistical analysis. MJK, VM and AFJR were involved in writing the manuscript. MJK, TRJ, VM and AFJR designed the experiments and interpreted the results. All authors read and approved the final manuscript.

## Supplementary Material

Additional file 1 Differentially expressed genes between B36 and N402 maltose-limited chemostat cultures. Click here for file

Additional file 2 Network map based on GO-enrichment analysis using the differentially expressed, induced, and repressed gene sets in B36/N402 chemostat cultures. Click here for file

Additional file 3 Enriched-GO terms using the differentially expressed, induced, and repressed gene sets in B36/N402 chemostat cultures. Click here for file

Additional file 4 Four higher-order categories of enriched-GO terms using the induced gene set in B36/N402 chemostat cultures. Click here for file

Additional file 5 Expression values of iron uptake genes. Click here for file

Additional file 6 Expression values of protease genes. Click here for file

Additional file 7 **Enriched-GO terms from the comparison between B36/N402 *versus *maltose/xylose.** Click here for file

Additional file 8 Primers used in qPCR and RT PCR. Click here for file
