# Supplementary material for: The transcriptomic fingerprint of glucoamylase over-expression in Aspergillus niger
Source: BMC Genomics. 2012 Dec 13;13:701. doi: 10.1186/1471-2164-13-701 (PMC3554566; doi:10.1186/1471-2164-13-701)

Additional file 2; network maps; all differentially expressed gene set

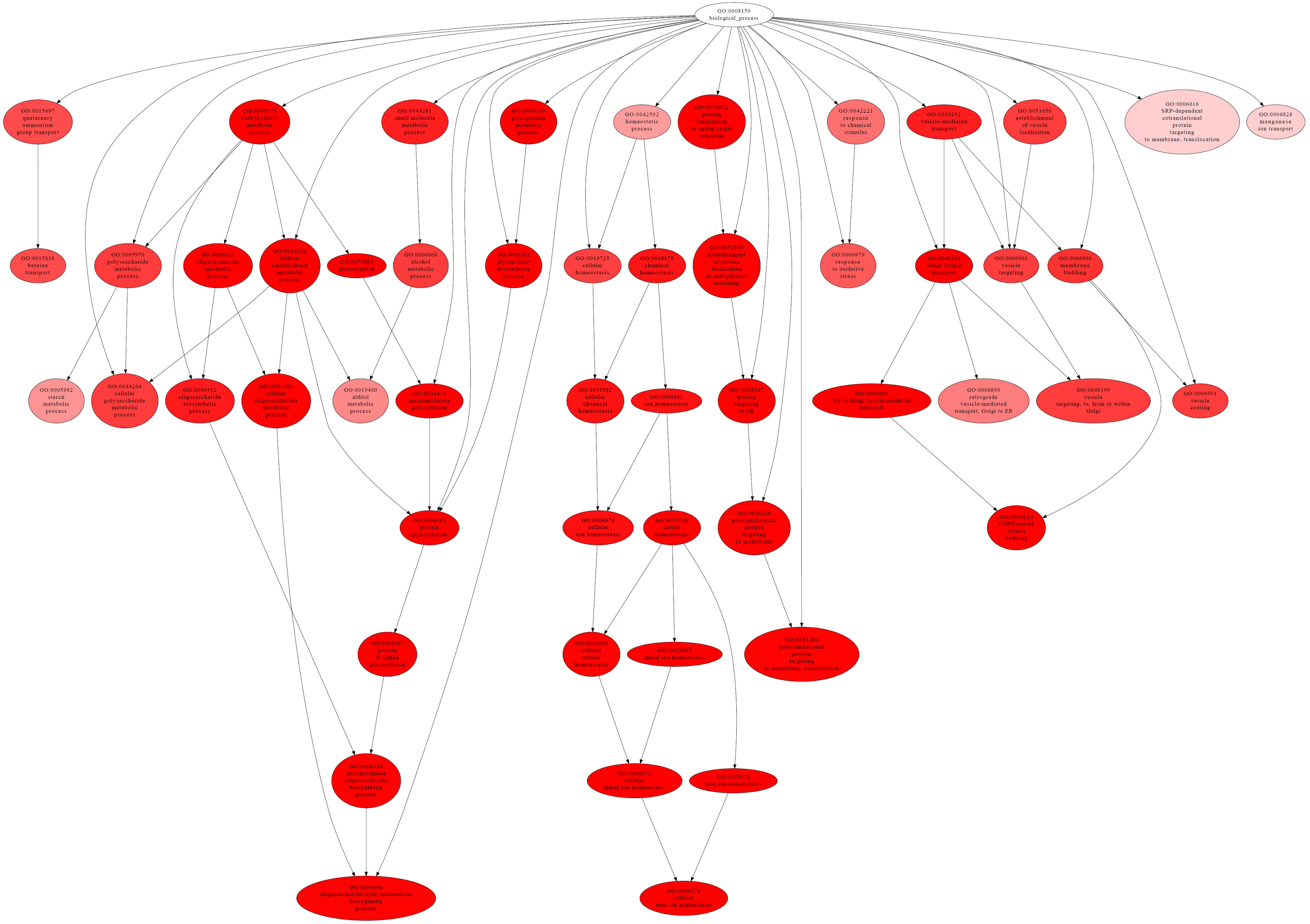

Additional file 2; network maps; up-regulated gene set

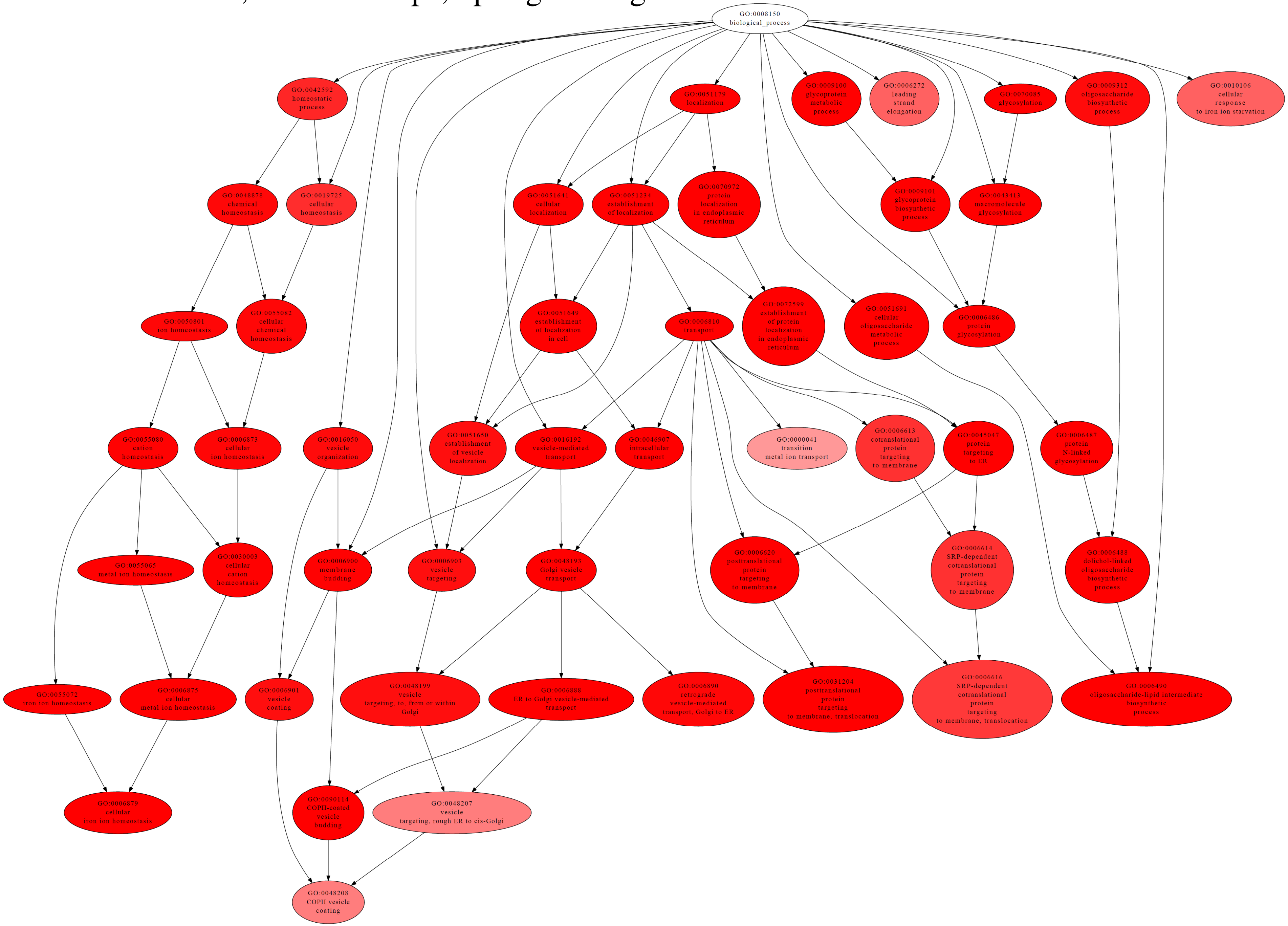

Additional file 2; network maps; down-regulated gene set

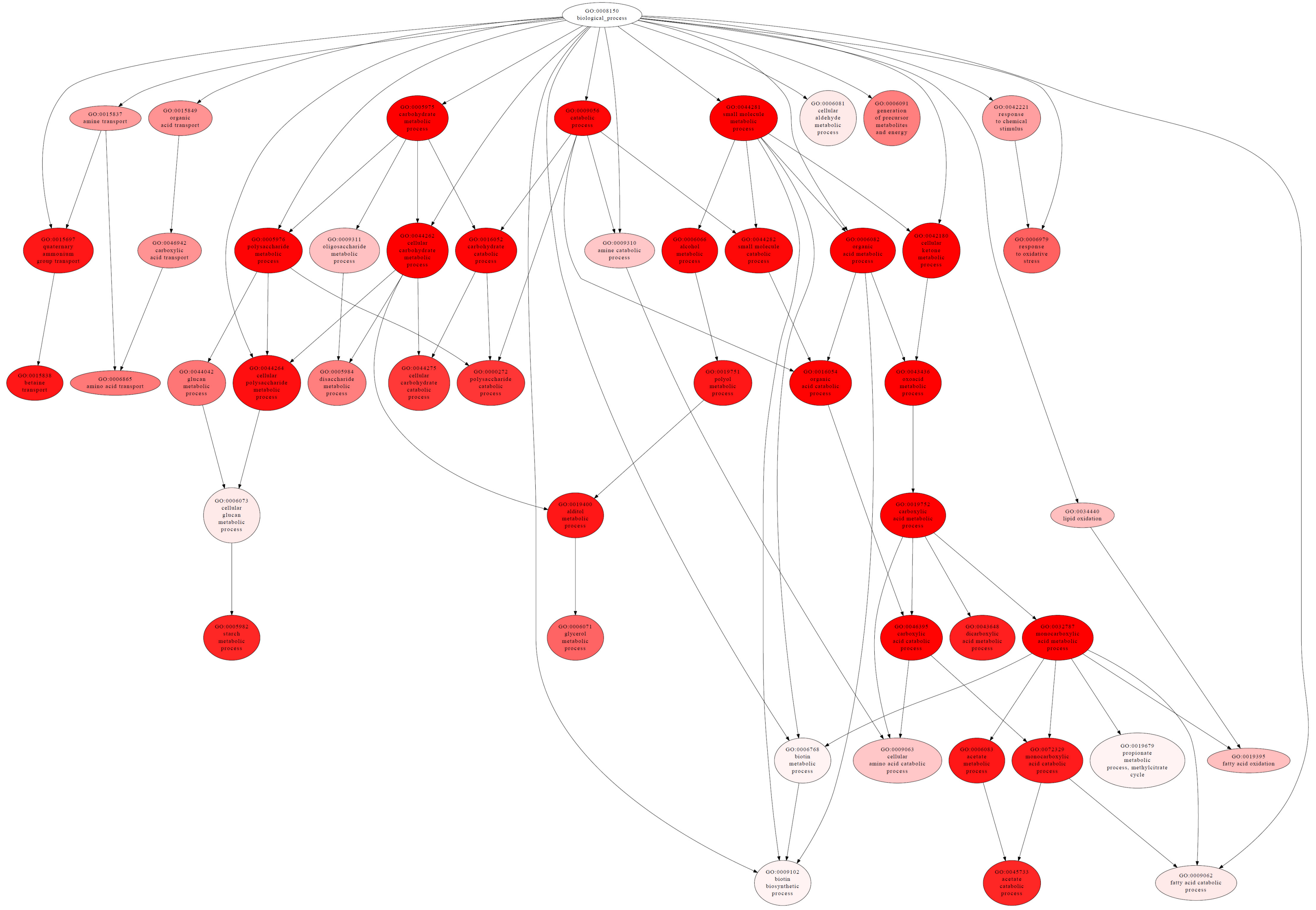

Supplement: Additional file 2 — Network map based on GO-enrichment analysis using the differentially expressed, induced, and repressed gene sets in B36/N402 chemostat cultures. [file 1471-2164-13-701-S2.pdf]
