# Supplementary material for: The transcriptomic fingerprint of glucoamylase over-expression in Aspergillus niger
Source: BMC Genomics. 2012 Dec 13;13:701. doi: 10.1186/1471-2164-13-701 (PMC3554566; doi:10.1186/1471-2164-13-701)
Supplement: Additional file 6 — Expression values of protease genes. [file 1471-2164-13-701-S6.docx]

**Additional File 6.** Expression values of protease genes.

| DSM code | | DSM annotation | fold difference B36/N402 | P | FDR |
| --- | --- | --- | --- | --- | --- |
| An01g00370 | strong similarity to aspergillopepsin apnS - Aspergillus phoenicis | | 0.54 | 4.96E-06 | 1.05E-04 |
| An02g07210^*^ | aspartic protease pepE - Aspergillus niger | | 0.97 | 4.64E-01 | 6.95E-01 |
| An04g01440^*^ | strong similarity to precursor of pepsin A3 - Homo sapiens | | 0.88 | 2.30E-02 | 9.44E-02 |
| An12g03300 | strong similarity to aspartic protease pr1 - Phaffia rhodozyma | | 0.60 | 7.07E-07 | 2.20E-05 |
| An13g02130 | strong similarity to aspartic proteinase Yps3 - Saccharomyces cerevisiae | | 2.54 | 7.67E-09 | 6.59E-07 |
| An14g04710 | aspartic proteinase aspergillopepsin I pepA - Aspergillus niger | | 0.03 | 7.77E-11 | 2.09E-08 |
| An15g06280^*^ | strong similarity to aspartic proteinase aspergillopepsin I pepA - Aspergillus niger [truncated ORF] | | 0.81 | 3.78E-03 | 2.31E-02 |
| An18g01320 | strong similarity to extracellular protease precursor Bar1 - Saccharomyces cerevisiae | | 1.29 | 1.02E-04 | 1.26E-03 |
| An03g01660^*^ | strong similarity to vacuolar aminopeptidase Y Ape3 - Saccharomyces cerevisiae | | 0.98 | 7.38E-01 | 8.79E-01 |
| An02g04690 | strong similarity to serine-type carboxypeptidase I cdpS - Aspergillus saitoi | | 0.09 | 1.37E-12 | 1.25E-09 |
| An03g05200 | strong similarity to carboxypeptidase S1 - Penicillium janthinellum | | 0.62 | 4.04E-07 | 1.41E-05 |
| An14g02150^*^ | strong similarity to serine-type carboxypeptidase precursor cpdS - Aspergillus phoenicis | | 0.95 | 2.50E-01 | 4.84E-01 |
| An08g04490 | endoprotease Endo-Pro - Aspergillus niger | | 0.37 | 3.42E-09 | 3.40E-07 |
| An12g05960 | strong similarity to dipeptidyl peptidase II DPPII - Rattus norvegicus | | 0.56 | 4.31E-08 | 2.63E-06 |
| An01g01750 | similarity to lysosomal protease CLN2 - Rattus norvegicus | | 0.33 | 1.80E-08 | 1.26E-06 |
| An03g01010 | strong similarity to lysosomal pepstatin insensitive protease CLN2 - Homo sapiens | | 0.54 | 4.43E-07 | 1.51E-05 |
| An06g00190 | strong similarity to lysosomal pepstatin insensitive protease CLN2 - Homo sapiens | | 0.26 | 2.48E-09 | 2.56E-07 |
| An08g04640 | strong similarity to hypothetical lysosomal pepstatin insensitive protease CLN2 - Canis lupus | | 0.59 | 1.59E-07 | 7.08E-06 |
| An14g02470 | strong similarity to protein PRO304 from patent WO200104311-A1 - Homo sapiens | | 0.46 | 1.97E-04 | 2.12E-03 |

DSM code: ORF identifier in *A. niger* CBS 513.88 genome sequence [[3](#_ENREF_4)]. The expression values of genes encoding proteases that were identified in the extracellular medium [[8](#_ENREF_8)]. ^*^ indicates non differentiually expressed genes.
