# Supplementary material for: The transcriptomic fingerprint of glucoamylase over-expression in Aspergillus niger
Source: BMC Genomics. 2012 Dec 13;13:701. doi: 10.1186/1471-2164-13-701 (PMC3554566; doi:10.1186/1471-2164-13-701)
Supplement: Additional file 8 — Primers used in qPCR and RT PCR. [file 1471-2164-13-701-S8.doc]

**Additional file 8**. Primers used in qPCR and RT PCR.

| Gene | Forward/reverse primers (5' 🡪 3') |
| --- | --- |
| *glaA* | GCCGATGGCTTCGTCTCTAT |
| *glaA* | GTCGTATTGCTCGGACATGG |
| H2B | CTCGAAACTTGCCGCTTACA |
| H2B | ACTTCGTGACAGCCTTGGTG |
| Cox5 | CATCTCGAATCCCACACTCG |
| Cox5 | TAGGCGGCCTTCTTCTCCT |
| HacA | CTTCTCCTACCCTAACTCCT |
| HacA | TCAAAGAGAGAGAGGGCA |
